# A pseudotumoral form of Crohn's disease: A case report and review of the literature

**DOI:** 10.1002/ccr3.3875

**Published:** 2021-01-27

**Authors:** Meriem Sabbah, Ghada Gharbi, Raja Jouini, Dhafer Haddad, Norsaf Bibani, Chiraz Chamakhi, Dalila Gargouri

**Affiliations:** ^1^ Department of Gastroenterology Habib Thameur Hospital Tunis Tunisia; ^2^ Faculty of Medicine of Tunis University of Tunis El Manar Tunis Tunisia; ^3^ Department of Pathology Habib Thameur Hospital Tunis Tunisia; ^4^ Department of Surgery Habib Thameur Hospital Tunis Tunisia; ^5^ Department of Radiology Habib Thameur Hospital Tunis Tunisia

**Keywords:** Crohn's disease, pseudotumoral form

## Abstract

In front of a colonic tumor, the diagnosis of a pseudotumoral form of Crohn's disease must be considered. However, it is a rare form, especially when inaugural and a neoplasia must be eliminated before retaining the diagnosis.

## Graphical abstract

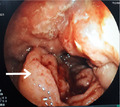

## 1 INTRODUCTION

The pseudotumoral form of Crohn's disease is rare, especially when inaugural. A neoplasia must be eliminated in front of a colonic tumor before retaining the diagnosis, hence the need of macrobiopsies or even pathological evidence on the operating specimen. A case of a 44‐year‐old patient in whom the diagnosis of inaugural Crohn's disease in its pseudotumoral form was retained on the data of the pathological examination of the operating specimen after a right hemicolectomy is reported. A review of cases presented in the literature is also performed.

## 2 CASE

A 44‐year‐old male patient, with history of appendectomy in 1994, operated twice for perianal abscesses in 2017 and 2018, consulted the emergency department for abdominal pain and subocclusive syndrome associated with deterioration in general condition with a weight loss of 10 kg.

Abdominal examination found a painful 4 cm soft mass in the right iliac fossa and biological findings revealed a biological inflammatory syndrome (WBC = 10880/mm^3^, CRP = 11.2 mg/dL), a hypochromic microcytic anemia at 11.8 g/dL, and hypoalbuminemia at 28 g/L. The tumor markers (carcinoembryonic antigen) were normal.

**FIGURE 1:**
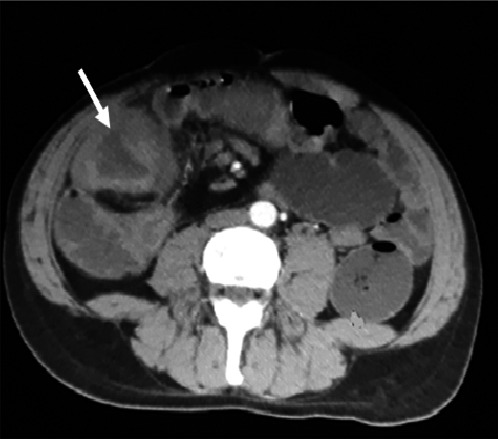
Longitudinal section of abdominal CT showing wall thickening of the right colon (arrow)

The abdomino‐pelvic CT scan with contrast injection showed a parietal circumferential thickening of the ascending colon and the cecum with a stenosing thickening near the right colic angle coming into contact with the segment VI of the liver and the last ilea loop with loss of the fatty border of safety with the latter. A significant infiltration of the intra‐abdominal fat in the right iliac fossa associated with multiple suspicious ganglia of the right mesocolon, as well as ganglia and lymphadenopathy of the root of the mesentery (Figure 1).

**FIGURE 2:**
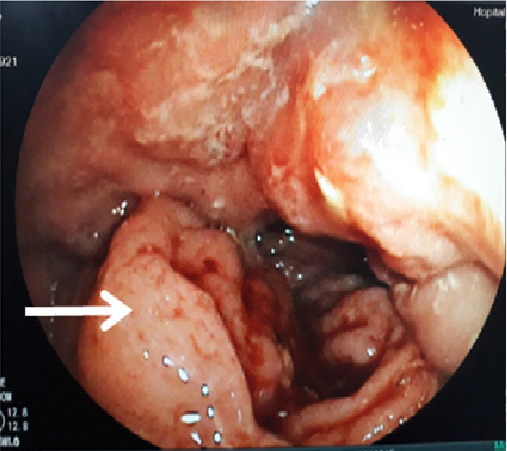
Colonoscopy: ulcerative‐budding process of the cecum (arrow)

The colonoscopy performed twice showed a stenosing ulcerative‐budding process of the right colon impassable by the colonoscope (Figure 2) and pathological examination of this process revealed erosive colitis with non specific chronic inflammatory changes and the absence of histological signs of malignancy.

The discrepancy between the endoscopic findings and the pathological examination, the possibility of colonic neoplasia, and the stenosing character of the colonic process prompted us to operate the patient on. Intraoperatively, we found a magma of inflammatory and fibrotic digestive loops formed by a meter of loops, the cecum, and the segment VI of the liver. There were also lymphadenopathies, and there was no individualized mass within this magma. The patient had a right hemicolectomy with a side to side ileocolic anastomosis.

**FIGURE 3:**
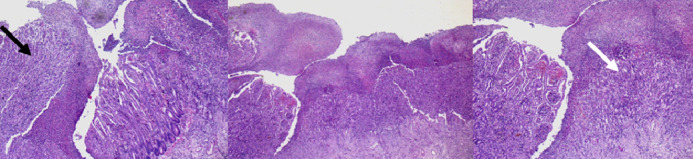
Pathological aspect: Ulcerated colic mucosa replaced by young fleshy buds (white arrow) and large inflammatory pseudopolyps (black arrow)

**FIGURE 4:**
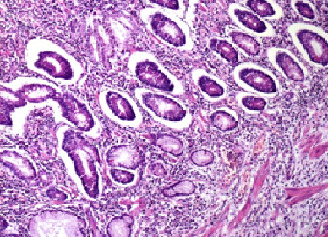
Pathological aspect of the nonulcerated mucosa showing signs of chronicity: architectural distortion and pseudopyloric metaplasia

The diagnosis of Crohn's disease was made on the data of pathological examination showing many mucosal ulcers sometimes narrow in "V" often reaching the mucous muscle, elsewhere broad exposing mucosal muscle. These ulcerations were often filled by a young fleshy bud with inflammatory pseudopolyps. Apart from ulcerations, the mucosa showed signs of chronicity associating architectural distortion and pseudopyloric metaplasia. The mucosecretion was little diminished. The chorion was very edematous with a dense, predominantly mononuclear inflammatory infiltrate. The submucosa was edematous with lymphoid follicular hyperplasia. The muscular tissue was little modified. There was also subserosal abscess, indicating the presence of fistulous paths. The serosa is lined with fibrino‐leukocyte coating. After a collegial opinion, the diagnosis was that of chronic ulcerative and stenotic segmental ileitis suggestive of Crohn's disease (Figures 3 and 4).

Currently, the patient is asymptomatic and treatment with immunomodulators (anti‐TNF alpha) is indicated because of the presence of a risk factor for postoperative recurrence (tobacco) as recommended by the European Crohn's Colitis Organization^1^ and the American gastroenterological association.^2^ He was prescribed infliximab 5 mg/kg every 8 weeks.

## 3 DISCUSSION

We report a case of Crohn's disease in its pseudotumoral form which remains an exceptional presentation of the disease. The main differential diagnosis in front of a colonic tumor is neoplasia which we must strive to eliminate, especially since Crohn's disease can degenerate into adenocarcinoma. Meanwhile, other differential diagnosis should also be considered as intestinal tuberculosis^3^, ^4^, ^5^, ^6^ and amoeboma,^7^ hence they can have the same clinical presentation and endoscopic findings as the Crohn's disease. Sometimes, the etiological diagnosis of an inflammatory pseudotumor of the colon remains unknown^8^ and the histological examination of the operating specimen is mandatory just to eliminate a neoplasia.

In fact, this pseudotumoral form of Crohn's disease has been already reported in the literature. Its clinical presentation is variable: abdominal mass like our case and the case of Stoica et al,^9^ obstruction and pseudo‐obstruction as our patient and other three cases reported by Fekih et al,^10^ weight loss like our case and the case of Maamouri et al,^8^ dysentery syndrome,^10^ bloody diarrhea,^11^, ^12^ rectal syndrome,^11^ rectal bleeding,^10^ abdominal pain, vomiting,^14^ and acute right iliac fossa.^10^

The abdomino‐pelvic CT scans performed in most of these patients with a colonic tumor were not contributory to the diagnosis of these pseudotumoral forms as they fail to differentiate them from colonic neoplasia. Thus, the diagnosis must be made by pathological examination.

In fact, histological examination of macrobiopsies of the process helped the diagnosis of Crohn's disease in only three cases of Maamouri et al,^8^ Mnif et al^13^ and Zouré Nogogna et al^14^ . In the other cases, patients underwent surgery and the diagnosis was made on pathological examination of the operating specimen.

In our patient case, we tried to obtain a histological confirmation to avoid surgery, as medical treatment can be enough in Crohn's disease. We performed biopsies of the colonic process twice but they were not contributory to the diagnosis. Hence, the patient was operated on to eliminate a neoplasia and have the definitive diagnosis.

Two of the patients in whom the diagnosis was made on macrobiopsies, clinical,^8^ and endoscopic^13^ remission were fulfilled under medical treatment (aminosalicylates^8^ and corticoids^13^).The third one has developed a painful mass of the right flank associated with a subocclusive syndrome under azathioprine and was thus operating on.^14^

The cases reported in the literature are summarized in Table 1.

**TABLE 1:** Review of the literature concerning pseudotumoral forms of Crohn's disease

Author and year	Number of cases reported	Age	Disease location	Time since diagnosis	Disclosure mode	Colonoscopy	pathology	treatment	Evolution
Stoica 1980^9^	1	42	Left colon	Inaugural	Abdominal mass and dysentery syndrome	NP	NP	Surgery	Recidivism
Maamouri 2011^11^	1	23	Rectal	Inaugural	Bloody diarrhea and weight loss	several budding lesions taking three‐quarters of the circumference, from 7 to 9 cm from the anal margin	Ulcerated rectal mucosa replaced by fibrin and leukocytes. The crypts and Lieberkühn glands were distorted, elongated, and branched with few cryptic abscesses. The chorion contained a dense inflammatory infiltrate, consisting mainly of lymphocytes and plasma cells	Suppositories of aminosalicylates	Good
Mnif 2013^13^	1	46	Right colon	Inaugural	Rectal bleeding	Three budding polypoid formations, friable on biopsy, of 5, 2 and 3 cm located respectively in the upper rectum, the left colic angle, and the right colic angle	Chronic segmental active mucosal inflammation with the presence of fissure ulcers, without pathological modification of the crypts. Diffuse edematous fibrosis of the wall, including lymphoid follicles and drafts of epithelioid granulomas without caseous or fibrinoid necrosis	Oral corticosteroid therapy + suppositories of aminosalicylates	Good
Bouomrani 2016^12^	1	75	Right colon	Inaugural	bloody diarrhea, weight loss, and subocclusive syndrome	NP	A very inflammatory and thickened ileal and colonic mucosa with many aphthous ulcers and the presence of several types of benign hyperplastic widely ulcerated polyps replaced by a chronic granulation tissue rich in neovessels and polymorphous inflammatory cells. No signs of malignancy were detected	Surgery (right colectomy)	Good
Zouré Nogogna 2019^14^	1	44	Right colon	Inaugural	constipation, bloating, and vomiting	an ulcero‐budding and stenotic mass in the right colon	Mucosal lesions made of ulcerations on the surface with crypts and elongated, tortuous, and deformed glands associated with cryptitis lesions. The chorion was congestive with a dense polymorphic inflammatory infiltrate associating lymphocytes, plasma cells, and numerous neutrophilic and eosinophilic polynuclear cells. The submucosa was intact and there was no tumor infiltrate	Oral corticosteroid therapy + Azathioprine	Recidivism
Fekih 2013^10^	16	43	Right colon	Inaugural	Obstruction (9 patients) and pseudo‐obstruction (3 patients), fever and acute right iliac fossa (4 patients)	NP	No signs of malignancy	surgery	Good
Tamzaourte 2009^15^	8	38		Inaugural	Subobstruction, an abdominal distention and a weight loss	Endoscopic failure of irregular and circumferential tumor in 3 cases and an irregular stenosis tumor looking in 2 cases	No signs of malignancy	surgery	Good
Our case	1	44	Right colon	Inaugural	Subobstruction	Stenosing ulcerative‐budding process of the right colon impassable by the colonoscope	Erosive colitis with non specific chronic inflammatory changes and absence of histological signs of malignancy	Surgery (right colectomy)	Good

Abbreviation: NP, Not Precised.

## 4 CONCLUSION

The pseudotumoral form of Crohn's disease is exceptional and appears to have its own clinical, morphological, and progressive features. The clinical and the endoscopic presentations are variable and the course of treatment is often favorable. It is probably not very aggressive compared to other forms of Crohn's disease. However, its characteristics remain to be defined.

## CONFLICT OF INTEREST

None declared.

## AUTHOR CONTRIBUTIONS

MS and GG: wrote the paper. GG: reviews the literature. RJ: contributes by the pathology pictures as well as the interpretation of figures. DH: operated the patient on. NB: referring Doctor. CC: contributes by the CT scan pictures. DG: The head of the gastroenterology department in the Hbib Thameu Hospital and contributes in the therapeutic decisions.

## ETHICAL APPROVAL

Patient personal data have been respected.
